# Tackling Antibiotic and Antifungal Resistance in Domestic Animals, Synanthropic Species, and Wildlife: A Global Health Imperative

**DOI:** 10.3390/antibiotics12111632

**Published:** 2023-11-17

**Authors:** Tamara Pasqualina Russo, Antonio Santaniello

**Affiliations:** Department of Veterinary Medicine and Animal Production, Federico II University of Naples, 80137 Naples, Italy; antonio.santaniello2@unina.it

Antibiotic resistance (ABR) and antifungal resistance (AFR) arise when microorganisms evolve mechanisms to resist pharmacological treatments. This resistance, often accelerated by the misuse and overuse of antibiotics and antifungals in both human and veterinary medicine, has far-reaching implications. The increasing prevalence of drug resistance is a pressing global health concern [1]; at present, the annual worldwide death count associated with antimicrobial resistance (AMR) is estimated to be 700,000. However, this number is predicted to experience a rapid increase, potentially reaching a distressing total of 10 million deaths per year by 2050 [2]. The efficacy of treatments for common bacterial infections is increasingly being compromised due to the global emergence and spread of antibiotic resistance [3]. The evolution of resistance is a multifaceted process influenced by the interplay of numerous biotic and abiotic factors. The key elements underpinning this dynamic include the rates at which resistant bacterial clones emerge and persist, the temporal and spatial gradients of antibiotics and other foreign substances, and the rates of transmission within human populations and between humans and various other sources such as animals, the environment, food, and so forth [4]. Similarly, pathogenic fungi exhibit numerous mechanisms of resistance to antifungal drugs, a phenomenon facilitated by their genetic adaptability and the versatility of their homeostatic responses to environmental stressors. This issue of escalating antifungal resistance is further exacerbated by the relatively limited availability of new antifungal agents [5].

In the context of domestic animals, synanthropic species, and wildlife, ABR and AFR pose significant challenges to animal health, biodiversity, and ecosystem stability. This Special Issue (SI) is dedicated to exploring this multifaceted phenomenon, and we are deeply grateful to all the authors who have contributed their insightful research. The collective body of work presented in this Special Issue underscores the complexity and urgency of the problem. The research presented herein highlights the widespread occurrence of multidrug-resistant microorganisms across a range of species and environments, emphasizing the potential public health implications. It also sheds light on the role of animals as potential reservoirs of AMR. A recurring theme across the studies is the importance of the One Health approach as a perspective recognizing the interdependence of human, animal, and environmental health and underscoring the need for a holistic approach to tackling health threats at the human–animal–environment interface [6].

Several studies in this issue highlight the role of various animal species, from birds and fallow deer to poultry and pigs, as potential reservoirs of antimicrobial-resistant bacteria (contributions 1–5). Additionally, a study conducted in a Belgian zoo identified the presence of azole-resistant *Aspergillus fumigatus* within a group of Humboldt penguins (contribution 6). These findings underscore the possibility that these microorganisms, sometimes pathogenic and resistant to one or more drugs, may spread across different environments, posing a significant public health concern. The research presented within this Special Issue also raises concerns about the presence of resistant bacteria in the food production industry, emphasizing the need for more effective screening and control measures. The potential for biofilm formation among resistant bacterial strains, which can facilitate the spread of resistance genes, is a particular area of concern (contribution 3).

This SI also includes research exploring potential alternatives to traditional antimicrobial agents, such as the use of *Bacillus* probiotics as animal growth promoters (contributions 7). These findings highlight the potential for innovative approaches to mitigate the impact of antimicrobial resistance. Moreover, the studies on poultry sperm quality highlight the need for more focus on the selection of an optimal combination and dose of antibiotics for poultry extenders (contribution 8). They emphasize the criticality of bacteriospermia in the poultry industry and highlight the need for more complex microbiological screening of semen samples designated for artificial insemination (contribution 9).

In conclusion, the critical role of ABR and AFR underscores the urgent need for action. It is our collective responsibility to tackle this global health challenge to safeguard the health of humans, animals, and our shared environment. The condition of the environment, along with its alterations and processes, plays a pivotal role in diseases mediated by animals. Anthropogenic stressors, including alterations in land use, loss of biodiversity, climate change, and pollution, further influence the role the environment plays at the interface of human and animal health [7]. Changes in land use, particularly regarding agriculture, also impact AMR. Antimicrobial agents are frequently overused and misused in various sectors, including human and veterinary medicine, farming, and industrial settings. This results in the accumulation of antibiotic residues, bacteria, antimicrobial-resistant metabolites, and AMR genes in wastewater, agricultural fields, and agricultural runoff [8,9].

We extend our sincere thanks to all the authors for their invaluable contributions to this Special Issue. Their work not only helps toward advancing our understanding of antimicrobial resistance but also underscores the urgent need for concerted, interdisciplinary efforts to address this global health challenge. We hope that this collection of studies will inspire further research and action in this critical area.
